# Sacubitril Does Not Exert Proarrhythmic Effects in Combination with Different Antiarrhythmic Drugs

**DOI:** 10.3390/ph18020230

**Published:** 2025-02-08

**Authors:** Christian Ellermann, Carlo Mengel, Julian Wolfes, Felix K. Wegner, Benjamin Rath, Julia Köbe, Lars Eckardt, Gerrit Frommeyer

**Affiliations:** Department of Cardiology II (Electrophysiology), University Hospital Münster, Albert-Schweitzer-Campus 1, 48149 Münster, Germany

**Keywords:** drug-induced proarrhythmia, Langendorff, arrhythmia, torsade de pointes, safety electrophysiology, ventricular tachycardia

## Abstract

**Background:** Previous studies suggest a direct effect of sacubitril on cardiac electrophysiology and indicate potential arrhythmic interactions between sacubitril and antiarrhythmic drugs. Therefore, the aim of this study was to explore the electrophysiologic effects of combining sacubitril with the antiarrhythmic drugs d,l-sotalol and mexiletine in isolated hearts. **Methods and results:** A total of 25 rabbit hearts were perfused using a Langendorff setup. Following baseline data collection, hearts were treated with mexiletine (25 µM, 13 hearts) or d,l-sotalol (100 µM, 12 hearts). Monophasic action potential demonstrated an abbreviation of action potential duration (APD_90_) after administration of mexiletine. Spatial dispersion of repolarization remained unchanged after mexiletine treatment, whereas effective refractory periods (ERP) were significantly prolonged. D,l-sotalol prolonged cardiac repolarization and amplified spatial dispersion. Further infusion of sacubitril (5 µM) led to a significant reduction in APD_90_ and ERP in the mexiletine group. In the d,l-sotalol group, additional administration of sacubitril shortened cardiac repolarization duration without affecting spatial dispersion. No proarrhythmic effect was observed after mexiletine treatment as assessed by a predefined pacing protocol. Additional sacubitril treatment did not increase ventricular vulnerability. When potassium concentration was reduced, 30 episodes of torsade de pointes tachycardia occurred after d,l-sotalol treatment. Additional sacubitril treatment significantly suppressed torsade de pointes tachycardia (eight episodes) in the d,l-sotalol-group. **Conclusions:** In class IB- and class III-pretreated hearts, sacubitril shortened refractory periods and cardiac repolarization duration. The combination of sacubitril with the antiarrhythmic drugs d,l-sotalol and mexiletine demonstrates a safe electrophysiologic profile and sacubitril reduces the occurrence of class III-related proarrhythmia, i.e., torsade de pointes tachycardia.

## 1. Introduction

Sacubitril is a neprilysin inhibitor and, in combination with valsartan, is recommended for the treatment of heart failure with reduced ejection fraction to reduce morbidity and mortality [1]. In PARADIGM-HF (Prospective Comparison of ARNI With ACEI to Determine Impact on Global Mortality and Morbidity in Heart Failure), sacubtril/valsartan reduced the risk of ventricular arrhythmias (VAs) with a hazard ratio of 0.76 [2]. These beneficial effects are mostly attributed to structural remodelling. However, previous studies suggest competing direct electrophysiological effects of sacubitril that may contribute to the antiarrhythmic effects of sacubitril [3]. Patients with structural heart disease with reduced ejection fraction are at risk of VAs and often need specific antiarrhythmic treatment. In the case of amiodarone and ablation refractory arrhythmias or intolerability to amiodarone, there is a need for other (additive) antiarrhythmic agents.

Mexiletine is a class IB antiarrhythmic agent that was developed in the late 1960s and is used for the treatment of ablation or drug refractory ventricular tachycardia and fibrillation [4]. Mexiletine is also recommended in long QT syndrome type 3 patients to shorten the QT_c_ interval and to reduce arrhythmias [5]. Of note, mexiletine also has beneficial effects in high-risk patients with long QT syndrome type 2 in addition to conventional therapies [6]. D,l-sotalol is a class III antiarrhythmic drug which is established as pharmacotherapy for the treatment of VAs in patients with moderate structural heart disease [4,5]. However, drug-induced proarrhythmia is a feared side effect due to the pronounced repolarization-prolonging properties of d,l-sotalol [4].

Recent case reports indicate potential fatal arrhythmic effects after combining sacubitril/valsartan with antiarrhythmic drugs: Weir et al. [7] reported two cases of patients on mexiletine who suffered VAs shortly after switching to sacubitril/valsartan. In both patients, no further arrhythmias were observed after cessation of sacubitril/valsartan or mexiletine, respectively. These cases raised awareness of the potential hazardous effects of a co-prescription of sacubitril/valsartan with antiarrhythmic drugs. Thus, the purpose of this study was to investigate the electrophysiological effects of a combination therapy of the neprilysin inhibitor sacubitril with the class IB-antiarrhythmic agent mexiletine and the class III drug d,l-sotalol.

## 2. Results

### 2.1. Electrophysiologic Effects of the Combination of Mexiletine and Sacubitril

**Figure 1:**
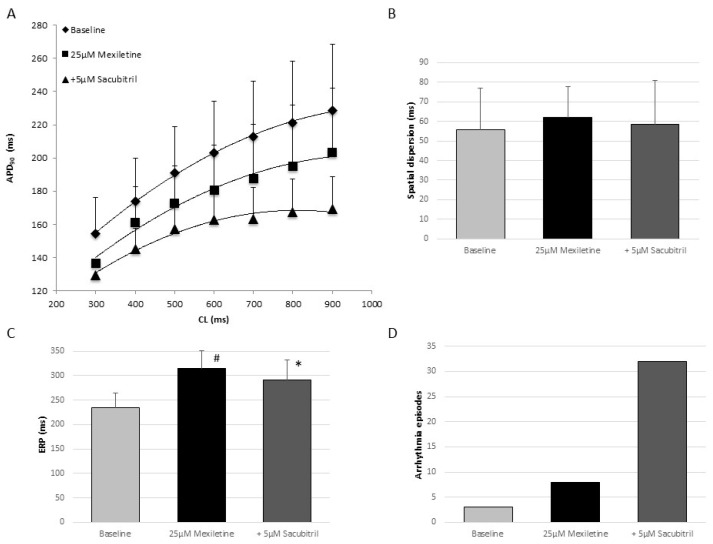
(**A**) Cycle length-dependent action potential durations at 90% of repolarization (APD_90_) under baseline conditions (◆), following perfusion with 25 µM mexiletine (■) and after additional administration of 5 µM sacubitril (▲). (**B**–**D**) Impact of 25 µM mexiletine and additional infusion of 5 µM sacubitril on spatial dispersion of repolarization (**B**), effective refractory periods (ERP, (**C**)), and the occurrence of ventricular arrhythmias induced by programmed ventricular stimulation (**D**). (# = *p* < 0.05 compared to baseline conditions; * = *p* < 0.05 compared to mexiletine).

Administration of mexiletine led to a significant abbreviation of APD_90_ (baseline: 198 ± 31 ms; mexiletine: 177 ± 30 ms, *p* < 0.01; Figure 1A) without altering the QT interval (baseline: 320 ± 27 ms; mexiletine: 331 ± 38 ms, *p* = ns). Further administration of sacubitril reduced APD_90_ (156 ± 17 ms, *p* < 0.01) and the QT interval (319 ± 44 ms, *p* < 0.01).

Spatial dispersion of repolarization remained stable after mexiletine treatment (baseline: 56 ± 21 ms; mexiletine: 62 ± 16 ms, *p* = ns; Figure 1B). Further perfusion with sacubitril did not change spatial dispersion (58 ± 22 ms, *p* = ns). As expected, sodium channel inhibition led to a prolongation of effective refractory periods (baseline: 234 ± 30 ms; mexiletine: 314 ± 37 ms, *p* < 0.01; Figure 1C), which was (partly) reversed by sacubitril (sacubitril: 291 ± 40 ms, *p* < 0.01). Mexiletine was not proarrhythmic in this study (eight episodes vs. three episodes under baseline conditions, *p* = ns; Figure 1D). Further sacubitril infusion did not significantly increase ventricular vulnerability (32 episodes, *p* = 0.06). No triggered activity was observed under hypokalemic conditions.

### 2.2. Electrophysiologic Effects of the Combination of D,l-Sotalol and Sacubitril

**Figure 2:**
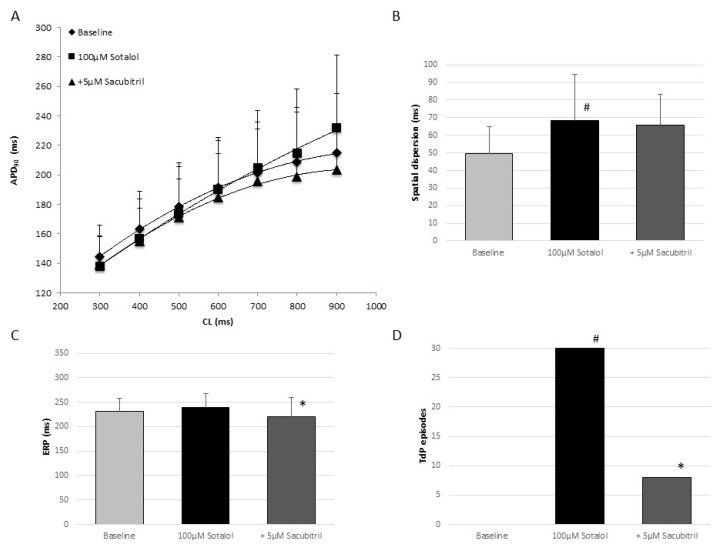
(**A**) Cycle length-dependent action potential durations at 90% of repolarization (APD_90_) under baseline conditions (◆), following perfusion with 100 µM d,l-sotalol (■) and after additional administration of 5 µM sacubitril (▲). (**B**–**D**) Impact of 100 µM d,l-sotalol and additional infusion of 5 µM sacubitril on spatial dispersion of repolarization (**B**), effective refractory periods (ERP, (**C**)), and the occurrence of torsade de pointes tachycardia (TdP) under hypokalemic conditions (**D**). (# = *p* < 0.05 compared to baseline conditions * = *p* < 0.05 compared to d,l-sotalol).

Inhibition of I_Kr_ by d,l-sotalol did not change APD_90_ (baseline: 186 ± 32 ms; d,l-sotalol: 187 ± 35 ms, *p* = ns; Figure 2A) but prolonged the QT interval (baseline: 293 ± 36 ms; d,l-sotalol: 317 ± 39 ms, *p* < 0.01) and augmented spatial dispersion of repolarization (baseline: 49 ± 16 ms; d,l-sotalol: 68 ± 26 ms, *p* < 0.01; Figure 2B). Effective refractory periods were not altered (baseline: 231 ± 27 ms; d,l-sotalol: 239 ± 29 ms, *p* = ns; Figure 2C). Additional treatment of sacubitril reduced APD_90_ (178 ± 33 ms, *p* < 0.05), the QT interval (304 ± 46 ms, *p* < 0.01), and effective refractory periods (221 ± 39 ms, *p* < 0.01). Spatial dispersion of repolarization was not affected (66 ± 18 ms, *p* = ns). Perfusion with a hypokalemic solution led to 30 episodes of torsade de pointes in the presence of d,l-sotalol (0 episodes under baseline conditions, *p* < 0.05; Figure 2D). Of interest, sacubitril significantly suppressed torsade de pointes tachycardia (eight episodes, *p* < 0.05). Early afterdepolarizations were observed in 8 out of 12 hearts in the presence of d,l-sotalol only and in 6 out of 12 hearts after additional perfusion with sacubitril.

## 3. Discussion

This study sought to determine the electrophysiologic effect of combining sacubitril with two different antiarrhythmic drugs, mexiletine and d,l-sotalol. The key findings of this study were as follows:

(1) Sacubitril abbreviates cardiac repolarization duration in mexiletine-pretreated hearts without amplifying the heterogeneity of cardiac repolarization.
(2) No significantly increased proarrhythmia could be determined with the simultaneous administration of mexiletine and sacubitril. However, a tendency towards more VAs was observed with the combination of both drugs.
(3) Sacubitril reduced the duration of cardiac repolarization in the presence of the class III-antiarrhythmic drug d,l-sotalol. Thereby, sacubitril exerted significant antiarrhythmic effects.

### 3.1. Combination of Mexiletine and Sacubitril

As anticipated for a class IB antiarrhythmic, sodium channel-inhibiting agent, administration of mexiletine led to a significant decrease of action potential duration, accompanied by a pronounced prolongation of effective refractory periods. Shortening of action potential duration in the presence of prolonged effective refractory periods results in an amplification of post-repolarization refractoriness, which is regarded as a key mechanism of its antiarrhythmic action [8,9]. In line with our current experiments, mexiletine shortened APD_90_ in guinea pig papillary muscles [10], probably mediated by an inhibition of I_Na,L_ [11]. Notably, in experimental models of acquired long QT syndromes, types 2 and 3, mexiletine demonstrated antiarrhythmic properties by reducing the spatial heterogeneity of repolarization and suppressed ventricular arrhythmias in a short QT syndrome model by prolonging refractory periods [12]. Accordingly, mexiletine is recommended for the pharmacological treatment of long QT syndrome type 3 according to current guidelines and also appears to show promising effects in long QT syndrome type 2 [5,6].

Additional administration of sacubitril further abbreviated cardiac repolarization duration and shortened effective refractory periods. Of note, spatial dispersion remained stable. An abbreviation of repolarization duration induced by sacubitril has been observed earlier by our group [3]. The underlying mechanism has not yet been elucidated. However, a sacubitril-mediated increase in I_Ks_ or I_Kr_ or a decrease of I_Na,L_ or I_Ca_ may account for the present observations [3]. The block of I_Ca_ seems of particular interest, as its block with verapamil has been shown to suppress torsade de pointes [13,14]. Though not as well recognized as QT prolongation-associated proarrhythmia, abbreviation of cardiac repolarization might also be a possible indicator for an increased proarrhythmic risk [15]. This is of particular interest since the combination of two drugs that influence cardiac repolarization duration may potentiate proarrhythmia by interfering with different transmembrane ion currents [16].

In the present study, no proarrhythmia was observed following mexiletine treatment. Additional infusion of sacubitril led to numerically more episodes of VAs. However, the results did not meet the threshold for statistical significance (*p* = 0.06). This borderline *p*-value makes it challenging to draw definitive conclusions. It is possible that additional experiments could yield significant results regarding inducible arrhythmias. However, based on numerous experimental studies conducted by our group, one would have expected to observe significant effects with the number of hearts studied if clinically relevant effects were present.

Although the present study does not show a significant proarrhythmic effect of the combination of mexiletine and sacubitril, one may speculate that their combined repolarization-abbreviating effect may explain the reported cases of arrhythmias [7]. In addition, the sacubitril-induced abbreviation of refractory periods can facilitate premature excitation and re-entry, thereby augmenting the risk for arrhythmias [17].

### 3.2. Combination of D,l-Sotalol and Sacubitril

In the present study, d,l-sotalol infusion had a significant proarrhythmic effect, with 30 episodes of torsade de pointes observed in the presence of a low potassium concentration. D,l-sotalol-induced proarrhythmia is a commonly known side effect mediated by a prolongation of action potential duration coupled with an augmented spatial dispersion of repolarization [18]. These effects have been demonstrated in the same experimental setup using both failing and non-failing rabbit hearts [19]. D,l-sotalol prolonged the QT interval and action potential duration, and increased the spatial dispersion of repolarization. D,l-sotalol induced torsade de pointes in 50% of non-failing hearts and in 89% of failing hearts [19].

Further treatment with sacubitril abbreviated cardiac repolarization duration, thereby reducing susceptibility to triggered activity. These findings are in line with a previous study in which acute sacubitril treatment reversed the prolongation of repolarization and subsequent proarrhythmia induced by administration of erythromycin as a different I_Kr_-inhibitor [3]. These experimental findings are in accordance with clinical data demonstrating that the normalization of the QT interval reduces the arrhythmic risk in patients with inherited long QT syndrome [20]. This also applies to acquired forms of the QT syndrome as greater QT interval prolongation increases the risk of drug-induced arrhythmias [21].

The results of this study suggest beneficial effects of sacubitril not only in (drug-induced) QT prolongation but also in heart failure, which can pathophysiological be considered as “mild manifestation” of long QT syndrome due to a reduced repolarization reserve [3,19,22]. This is of particular interest since the potential hazardous effects of d,l-sotalol are amplified in failing hearts, leading to a more pronounced increase of APD_90_ and spatial dispersion and an increase of triggered arrhythmias compared to healthy hearts [19]. Sacubitril might counteract these adverse effects and protect failing hearts from d,l-sotalol-related proarrhythmia. To prove these theoretical considerations, additional experiments in failing hearts are required. One possible approach would be to induce heart failure by rapid ventricular pacing, leading to a reduction in ejection fraction and heart failure symptoms [19], and then apply the same experimental protocol to these failing hearts.

Given sacubitril’s ability to shorten repolarization duration in various models of acquired long QT syndrome, sacubitril is a promising pharmacological option for the complementary treatment of long QT syndrome or arrhythmias associated with heart failure. In this context, sacubitril has shown a favourable safety profile in large contemporary heart failure trials [2,23,24]. However, to date, no clinical trials have been published assessing the safety profile of sacubitril in patients with long QT syndrome.

## 4. Materials and Methods

The experimental protocol was authorized by the local animal care committee (Landesamt für Natur, Umwelt und Verbraucherschutz Nordrhein-Westfalen, Germany; file number: 81-02.05.50.21.004) and conducted in compliance with the Guide for the Care and Use of Laboratory Animals published by the US National Institutes of Health (NIH Publication No. 852-3, revised 1996) as well as the ARRIVE guidelines. In this study, no randomization was performed as each heart acted as its own control. The sample size was determined based on prior studies from our group with similar expected effect size. No animals were excluded from this study.

### 4.1. Experimental Setup

The experimental Langendorff setup has been previously described [3]. In short, 25 hearts of female *New Zealand white rabbits*, aged 14–24 weeks, were retrogradely perfused utilizing a Langendorff setup (Hugo Sachs Elektronik, Medical Research Instrumentation, March Hugstetten, Germany). AV node ablation was performed employing surgical tweezers. Hearts perfused at a constant flow (52 mL/h) and pressure (90 mmHg). A modified Krebs–Henseleit buffer (NaCl 118 mM, NaHCO_3_ 24.88 mM, D-glucose 5.55 mM, KCl 4.70 mM, Na-pyruvate 2 mM, CaCl_2_ 1.80 mM, KH_2_PO_4_ 1.18 mM, MgSO_4_ 0.83 mM) was warmed, oxygenated (95% O_2_, 5% CO_2_), and served as perfusate. All electrolytes and drugs except d,l-sotalol were purchased from Sigma Aldrich (Steinheim, Germany). D,l-sotalol was acquired from Carinopharm GmbH (Eime, Germany). Seven specifically designed catheters were attached epicardially; one further catheter was introduced into the left ventricle to obtain epi- and endocardially recorded monophasic action potentials employing an electrophysiology recording system (Bard, Lab System Pro EP Recording System, Salt Lake City, UT, USA). At the same time, a volume-conducted 12-lead ECG was recorded.

### 4.2. Experimental Protocol

**Figure 3:**
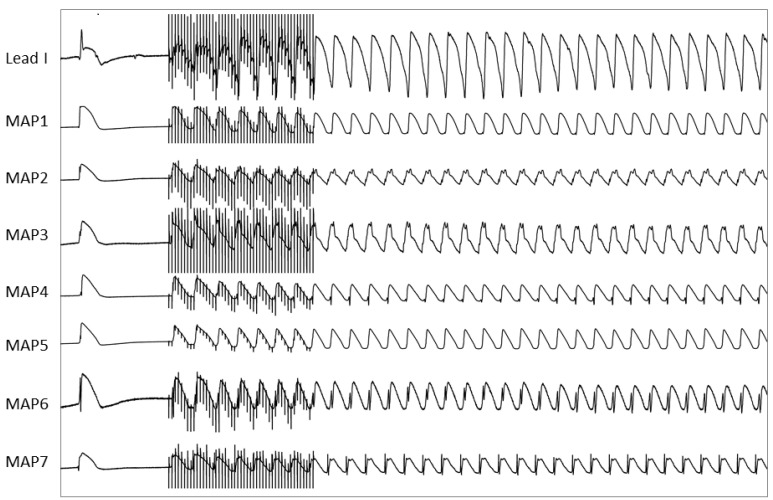
Train of burst pacing with induction of a ventricular tachycardia under baseline conditions (MAP = monophasic action potential).

Hearts were paced (using the Universal Heart Stimulator UHS 20S, Biotronik, Berlin, Germany) at different cycle lengths (900–300 ms) to record cycle length-dependent action potential duration and QT intervals. Premature extrastimuli (S_2_ and S_3_) were delivered after a train of seven stimuli (900–300 ms; S_1_) to obtain effective refractory periods and to assess ventricular vulnerability. In addition, burst pacing was utilized to provoke VAs (Figure 3). Thereafter, bradycardic hearts were perfused with a hypokalemic Krebs–Henseleit buffer (1.5 mM) to facilitate triggered activity. APD_90_ was determined by the time span between the fastest upstroke of the action potential and 90% of repolarization. Spatial dispersion of repolarization was defined as the difference between the longest and shortest APD_90_.

### 4.3. Experimental Groups

25 hearts were randomly allocated to two groups after generating baseline data. The first group (*n* = 13) was treated with the sodium channel blocking, class IB antiarrhythmic drug mexiletine (25 µM). 12 further hearts (*n* = 12) were perfused with the class III antiarrhythmic drug d,l-sotalol (100 µM), thereby inhibiting the potassium current I_Kr_. After completing the pacing protocol in the presence of these drugs, both groups were treated with sacubitril (5 µM) and the pacing protocol was repeated again. The researchers were not blinded during this study.

### 4.4. Statistics

Action potentials and electrograms were recorded using a multi-channel recorder and digitalized at a rate of 1 kHz with 12-bit resolution. Results are presented as mean ± standard deviation. Statistical analyses were conducted using SPSS Statistics for Windows (version 24.0). The effects of drugs on APD_90_, QT interval, spatial dispersion of repolarization, and effective refractory periods were analysed using a Wilcoxon signed rank test. *p* values < 0.05 were considered statistically significant.

## 5. Conclusions

Sacubitril in combination with antiarrhythmic drugs exhibits a safe electrophysiologic profile. In hearts treated with mexiletine or d,l-sotalol, sacubitril shortens effective refractory periods and cardiac repolarization duration without altering spatial dispersion of repolarization. Sacubitril demonstrated antiarrhythmic properties against d,l-sotalol-induced proarrhythmia. However, when combined with mexiletine, there was a tendency for increased proarrhythmia, suggesting that sacubitril may have a better safety profile when used with class III compared to class IB antiarrhythmic drugs. The findings from this study, along with our previous research [3], suggest that sacubitril has complex effects on cardiac ion channels, which warrant further investigation in the future.

## Data Availability

The datasets generated during and analysed during the current study are available from the corresponding author on reasonable request.
